# Chemical Basis of Prey Recognition in Thamnophiine Snakes: The Unexpected New Roles of Parvalbumins

**DOI:** 10.1371/journal.pone.0039560

**Published:** 2012-06-27

**Authors:** Maïté Smargiassi, Gheylen Daghfous, Baptiste Leroy, Pierre Legreneur, Gerard Toubeau, Vincent Bels, Ruddy Wattiez

**Affiliations:** 1 Department of Proteomics and Microbiology, Interdisciplinary Center of Mass Spectrometry (CISMa), University of Mons-UMONS, Mons, Belgium; 2 Département Ecologie et Gestion de la Biodiversité, Muséum National d’Histoire Naturelle, Paris, France; 3 EA 647, CRIS, Université de Lyon, Villeurbanne, France; 4 Department of Histology, University of Mons-Hainaut, Mons, Belgium; Russian Academy of Sciences, Institute for Biological Instrumentation, Russian Federation

## Abstract

Detecting and locating prey are key to predatory success within trophic chains. Predators use various signals through specialized visual, olfactory, auditory or tactile sensory systems to pinpoint their prey. Snakes chemically sense their prey through a highly developed auxiliary olfactory sense organ, the vomeronasal organ (VNO). In natricine snakes that are able to feed on land and water, the VNO plays a critical role in predatory behavior by detecting cues, known as vomodors, which are produced by their potential prey. However, the chemical nature of these cues remains unclear. Recently, we demonstrated that specific proteins–parvalbumins–present in the cutaneous mucus of the common frog (*Rana temporaria*) may be natural chemoattractive proteins for these snakes. Here, we show that parvalbumins and parvalbumin-like proteins, which are mainly intracellular, are physiologically present in the epidermal mucous cells and mucus of several frog and fish genera from both fresh and salt water. These proteins are located in many tissues and function as Ca^2+^ buffers. In addition, we clarified the intrinsic role of parvalbumins present in the cutaneous mucus of amphibians and fishes. We demonstrate that these Ca^2+^-binding proteins participate in innate bacterial defense mechanisms by means of calcium chelation. We show that these parvalbumins are chemoattractive for three different thamnophiine snakes, suggesting that these chemicals play a key role in their prey-recognition mechanism. Therefore, we suggest that recognition of parvalbumin-like proteins or other calcium-binding proteins by the VNO could be a generalized prey-recognition process in snakes. Detecting innate prey defense mechanism compounds may have driven the evolution of this predator-prey interaction.

## Introduction

Many predators have evolved highly specialized sensory systems to detect and locate their prey [1]. For example, barn owls have asymmetric ear openings that allow them to localize their prey by triangulation [2], bats have an echolocation system [3], raptors have a high visual acuity associated with two foveae [4], star-nosed moles have a unique mechanosensory organ [5] and sharks and rays have an electrostatic sense [6].

Snakes have the most highly developed vomeronasal organ of all vertebrates, and vomeronasal chemoreception is known to be the dominant sensory mode used by most snakes to detect and locate prey [7]–[9]. In natricine snakes, this sensory system plays an even more critical role in feeding behavior because vomeronasal cues are necessary and sufficient to recognize species-specific prey [10], [11] and elicit prey attack [12], [13]. Consequently, natricine snakes have been widely studied as a model system for investigating the genetic and environmental factors that influence chemoreceptive prey responses and dietary preferences [7], [8], [14]–[16]. However, little is known about the nature of the chemical cues involved. To date, only two chemoattractant molecules that elicit prey attack in natricine snakes have been identified: parvalbumins from the mucus extracts of the common frog (*Rana temporaria*) [9], [17], and electro-shock-induced protein (ES20) from the secretions of earthworms (*Lumbricus terrestris*) [18]–[20]. The chemoattractive properties of these proteins have been shown to be vomeronasally mediated and calcium dependent [9], [17], [20]. In the vomeronasal epithelium, ES20 binds to a G protein-coupled receptor [21], [22]. Parvalbumins are vertebrate-specific calcium-binding proteins that belong to the super family of the EF-hand proteins [23]. Interestingly, calcium also plays a crucial role in the chemoattractivity of parvalbumins [9]. Before the demonstration of parvalbumins in cutaneous mucus of *Rana temporaria* [9], these proteins were only known from muscular and nervous tissues where they function as calcium buffers [24], [25]. The extracellular localization of parvalbumins remains unknown, and the function of these proteins in the cutaneous mucus is unclear. We hypothesized that parvalbumins and other calcium-binding proteins play a key role in the chemical prey recognition mechanisms of thamnophiine snakes. Our study focused on three species of natricine snakes (*Thamnophis marcianus*, *Thamnophis sirtalis* and *Nerodia fasciata*) that are representative of the two major clades of new world natricine snakes. They share similar dietary habits, principally fish and amphibians [26]. This study was specifically designed to: 1) investigate the presence of parvalbumins in cutaneous mucus of two ecologically relevant prey of these snakes (*Lithobates catesbeianus* and *Pimephales promelas*) and two fish species (*Tanichthys albonubes* and *Osmerus eperlanus*) that are not part of the natural diet of these snakes 2) to establish the phylogenetic extent of this chemical based prey-predator relationship and to 3) to investigate the physiological significance of presence of parvalbumins in cutaneous secretions of fish and amphibians.

## Results

### Bioactivity of Protein Extracts from the Cutaneous Mucus

In this study, we showed the presence of chemoattractive proteins in the cutaneous mucus of the tested frogs and fish. First, protein extracts derived from the cutaneous mucus of the prey species (*Lithobates catesbeianus, Pimephales promelas* and *Osmerus eperlanus*) were submitted to unambiguous snake bioassays (Video S1). All of the extracts elicited an attack of the lure in all three of the thamnophiine snakes, suggesting that the crude extracts contain chemoattractive compounds (Table 1). After treatment with a nonspecific proteolytic enzyme (proteinase K), the crude extracts no longer triggered snake attacks, pointing to the crucial chemoattractive role of the proteins (Table 1).

**Table 1:** Bioassays of the snake protein extracts derived from the cutaneous mucus of the prey species.

	*Lithobates catesbeinus*	*Osmerus eperlanus*	*Pimephales promelas*
*Thamnophis marcianus*	8/8∶0/2	23/23∶0/5	3/3∶0/2
*Thamnophis sirtalis*	3/3∶0/2	19/19∶0/4	2/2∶0/2
*Nerodia fasciata*	2/2∶0/2	19/19∶0/5	2/2∶0/2

The chemoattractivity of the crude mucus extracts was assessed according to a standard “all-or-none” snake bioassay based on a non-biological lure coated with the tested sample as in [9]. The test was considered positive if the snake attacked the lure within 20 sec after the first lure-directed tongue flick. The test was considered negative in any other circumstances. A/B corresponds to the number of positive tests/total tests. X/Y corresponds to the number of positive tests/total tests after the proteinase K treatment.

### Characterization of Chemoattractants

**Figure 1:**
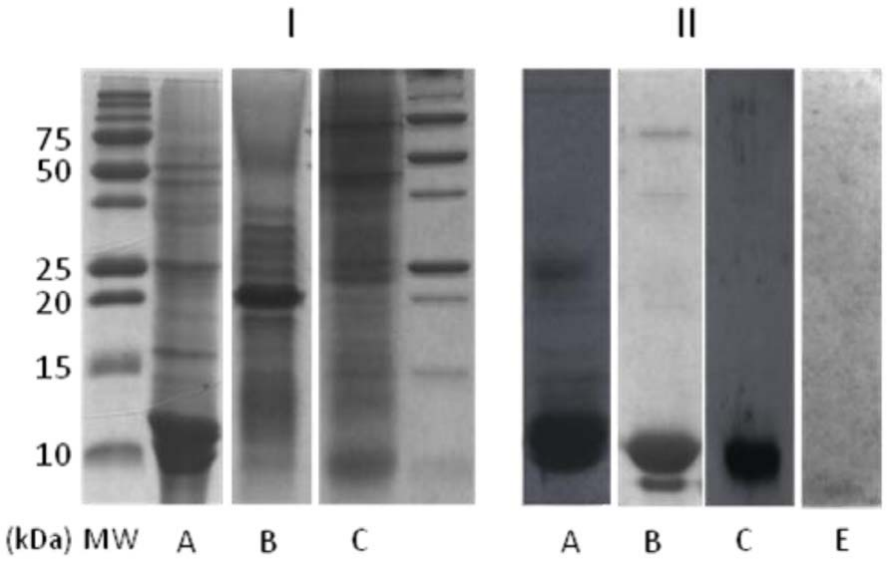
Characterization of the crude mucosal extracts of (a) *Osmerus eperlanus,* (b) *Lithobates catesbeianus* and (c) *Pimephales promelas*. I : the protein extracts from cutaneous mucus were obtained from aqueous washes of the prey according to the procedure described in [9]. The filtered extracts were extensively dialysed against water containing 7 mM β-MSH and then lyophilized. The lyophilized mucus extract was solubilized in a Laemmli sample buffer and subjected to SDS-PAGE. The SDS-PAGE were stained with coomassie blue reagent II : The presence of parvalbumins was confirmed by rabbit anti-α parvalbumin western blot analysis of protein extracts obtained from the different cutaneous mucus. The migration of the immunoreactive products was consistent with the molecular weight of the parvalbumin family (10–14 kDa). No labeling was observed in the presence of the anti-α parvalbumin antibodies pre-incubated with the purified parvalbumins from the crude mucosal extract of *Lithobates catesbeianus* (E).

**Figure 2:**
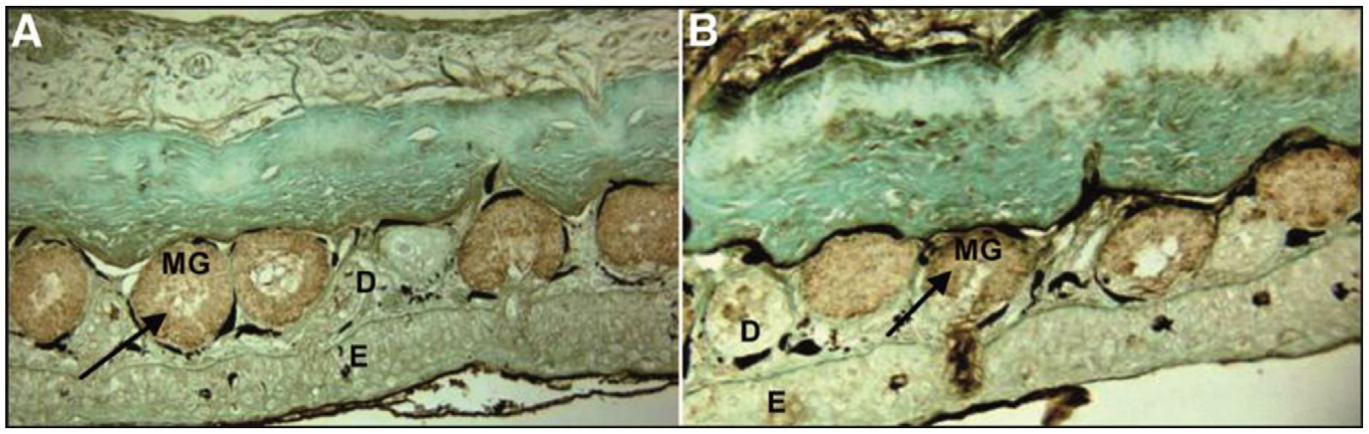
The immunohistochemistry of the amphibian skin sections. (**A**) A *Lithobates catesbeianus* skin section stained with an anti-mucosal α parvalbumin (*Rana temporaria*) antibody. (B) A *Lithobates catesbeianus* skin section stained with an anti-muscular α parvalbumin (*Lithobates catesbeianus*) antibody. Note the localization of the immunoreactive cells in the dermal mucous glands (arrows). D  =  Dermis, E  =  Epidermis, MG  =  Mucous Glands.

**Figure 3:**
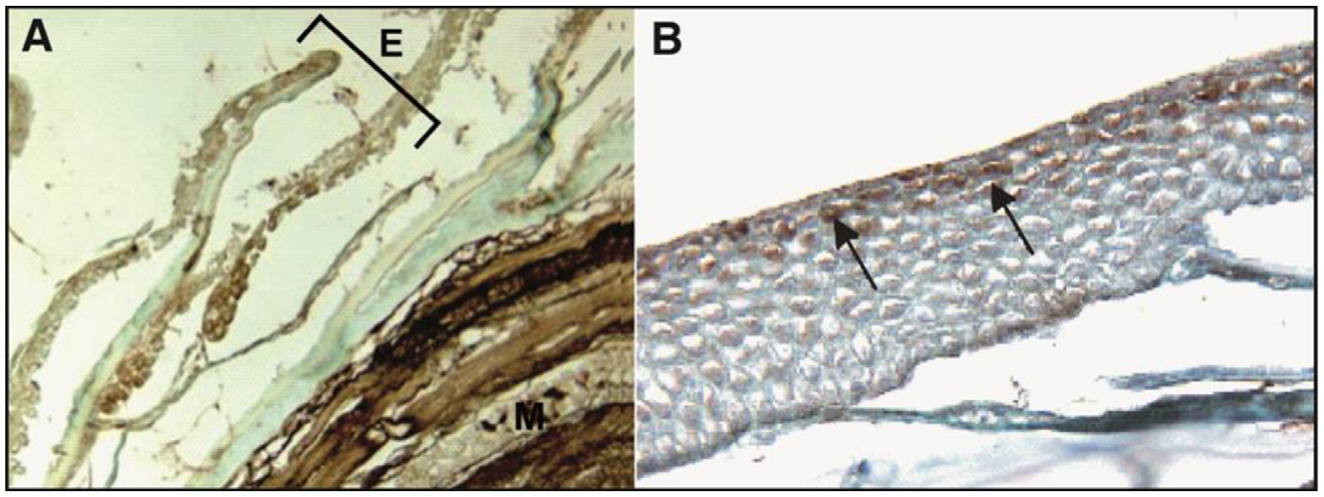
The immunohistochemistry of fish skin sections using an anti-muscular α parvalbumin (*Rana temporaria*) antibody. (A) *Pimephales promelas.* (B) *Tanichthys albonubes.* The arrows indicate positive epidermal mucous cells. E  =  Epidermis, M  =  Muscle.

The α and β parvalbumins, proteins found in the cutaneous mucus of the frog *Rana temporaria*, have been previously reported to elicit prey attacks in *Thamnophis marcianus* [9]. In our study, we reveal the physiological presence of parvalbumins or parvalbumin-like proteins in the skin mucus of all the tested amphibians and fish. For each cutaneous mucus protein extract, we demonstrated the presence of one or two specific anti-α parvalbumin immunoreactive proteins (Fig. 1). Moreover, we confirmed the presence of α and β parvalbumins in the 11 kDa and 10 kDa immunoreactive bands, respectively, of the *Osmerus eperlanus* crude extract using mass spectrometry analysis. However, this approach does not confirm the presence of parvalbumins in the crude extracts of the other tested species probably due to their low concentration. Consequently, the immunoreactive proteins present in the crude extracts of *Lithobates catesbeianus* and *Pimephales promelas* were considered to be parvalbumin-like proteins. Next, we examined the localization of α parvalbumin in skin sections. Specific α parvalbumin immunoreactivity was observed in the dermal mucous glands of *Lithobates catesbeianus* (Fig. 2), consistent with results for the dermal mucous glands of *Rana temporaria* [9]. The immunohistochemistry of the fish (*Pimephales promelas* and *Tanichthys albonubes*) skin sections also showed specific α parvalbumin immunoreactivity localized in the epidermal mucous cells (Fig. 3). Moreover, parvalbumin-like proteins were also found in the cutaneous mucus of a urodele amphibian, *Ambystoma mexicanum* (data not shown), suggesting that these proteins may be widely present among vertebrates with mucus-covered skin, such as fish and amphibians.

### Bioactivity of Purified Chemoattractants

**Figure 4:**
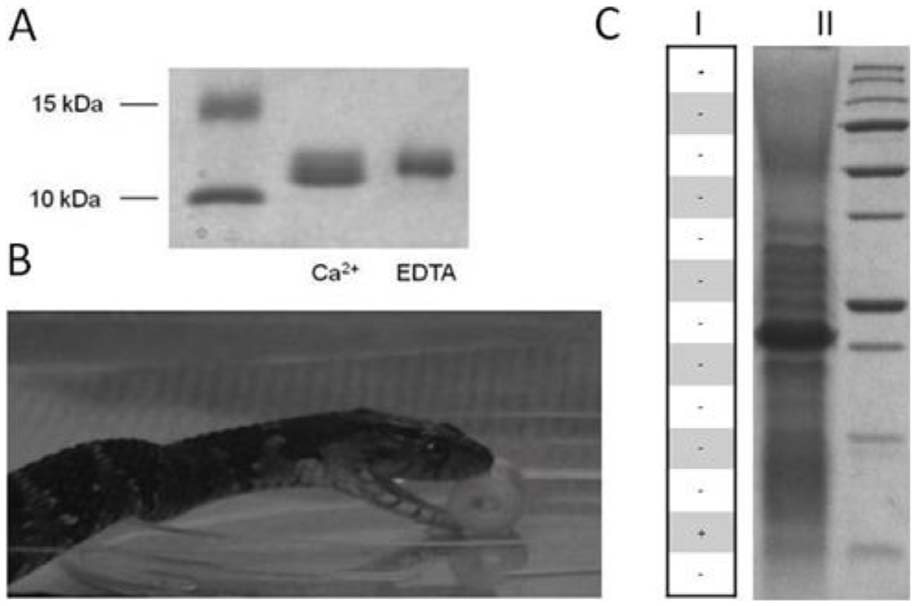
Chemoattractivity of parvalbumins. **A. The presence of calcium-complexed parvalbumins was monitored using SDS-PAGE.** *Osmerus eperlanus* α parvalbumin was purified according to the procedure described by Gosselin and Rey (1977) (34). Apo-parvalbumin was prepared by incubating the purified parvalbumin with a calcium chelating agent (2 mM EDTA) and was then dialysed to remove the EDTA. As has been observed for *Rana temporaria* parvalbumins, a mass shift was observed in the absence of calcium after the electrophoresis [9]. **B. The purified α parvalbumin showed a chemoattractant activity.** A non-biological lure (cooked macaroni) was coated with 30 µl of protein sample or sample buffer (control solution) and placed in the snake’s box in front of its shelter. The test was considered positive if the snake attacked the lure within 20 sec after the first lure-directed tongue flick. In any other case, the test was considered negative. **C. The chemoattractant activity of proteins from crude mucus extract** The lyophilized mucus extract obtained from *Lithobates catesbeianus* was solubilized in a Laemmli sample buffer and subjected to SDS-PAGE (C.II). 13 pieces of gel were cut as described in the figure (C.I) and proteins from these excised gel pieces were recovered by pulverizing and soaking them in 50 mM DTE for 24 hours under strong agitation at 4°C. The supernatants were collected, lyophilized and assessed according to a standard “all-or-none” snake bioassay based on a non-biological lure coated as described above. – Négative test, + Positive test.

The chemoattractive properties of the parvalbumins and parvalbumin-like proteins isolated from the cutaneous mucus of the three prey species were tested using a validated snake bioassay (Fig. 4B) [9]. All of them displayed chemoattractive activity in all three of the thamnophiine snakes (Table 2). In order to highlight the potential chemoattractivity of other proteins present in the cutaneous mucus of the prey, all the protein bands obtained by SDS-PAGE of each crude extract were submitted to snake bioassays. However, none of the other protein bands exhibited this activity (Fig. 4C). Immunological and behavioural results suggest that parvalbumins and parvalbumins-like proteins isolated from *Lithobates catesbeianus*, *Osmerus eperlanus* and *Pimephales promelas* crude extracts are necessary and sufficient to elicit prey attack. The parvalbumin threshold for eliciting prey-attack in natricine snakes was evaluated by testing different quantities of purified *Osmerus eperlanus* parvalbumins on *Nerodia fasciata*. Although an individual variability in the responses was observed in our bioassays, the threshold for eliciting lure attack was in the order of tens µg of for four out of five snakes. The lowest parvalbumin detection threshold recorded was on the order of µg (Table 3). Inter-individual variability for eliciting prey attack in responses to chemical stimuli from prey has been previously reported in natricine snakes [15], [16]. Among the factor accounting for this inter-individual variability, we should mention that geographic provenance/genetic [15], and ontogeny [16] have been shown to be related to feeding preference and thus to prey attack responses for a particular prey stimulus.

**Table 2:** Bioassays of the immunoreactive SDS-PAGE bands extracted from the cutaneous mucus of the prey species.

		*Thamnophis marcianus*	*Thamnophis sirtalis*	*Nerodia fasciata*
*Rana catesbeiana*	11 kDa band	2/2	2/2	ND
	14 kDa band	4/4	2/2	ND
*Osmerus eperlanus*	β parvalbumin	4/4	7/7	1/1
	α parvalbumin	5/5	4/4	14/14
*Pimephales promelas*	10 kDa band	5/5	2/2	1/1

The chemoattractivity of the purified proteins was assessed according to a standard “all-or-none” snake bioassay based on a non-biological lure coated with the tested sample as in [9]. The test was considered positive if the snake attacked the lure within 20 sec after the first lure-directed tongue flick. The test was considered negative in any other circumstances. A/B corresponds to the number of positive tests/total tests. ND  =  not determined.

**Table 3:** Parvalbumin threshold for eliciting prey-attack in *Nerodia fasciata*.

	*Snake 1*	*Snake 2*	*Snake 3*	*Snake 4*	*Snake 5*
Parvalbumin (ug)					
40,00	1/1	1/1	1/1	1/2	1/1
20,00	2/2	1/1	0/1	0/1	0/1
13,50	0/2	1/1	ND	ND	ND
10,00	0/1	1/1	ND	ND	ND
6,50	0/1	1/1	ND	0/1	0/1
3,25	0/1	1/1	ND	ND	ND
1,30	0/2	1/1	0/1	0/1	ND
0,65	ND	2/2	ND	ND	ND
0,50	ND	0/2	ND	ND	ND
0,10	ND	0/1	ND	ND	ND

The parvalbumin threshold for eliciting lure attack in *Nerodia fasciata* was assessed by using different quantities of purified parvalbumins in a bioassay. The threshold was shown to be in the tens of ug order of magnitude. Note that there is individual variation in the responses as individual 2 attacked lures coated with as low as 0.65 ug of parvalbumins. ND: response not determined.

### Calcium Dependence of Parvalbumins Bioactivity

Calcium has been reported to be required for ES20, an electro shock-induced protein found in the secretions of earthworms and that is involved in garter snake chemoattraction [19]–[21]. We showed that calcium also plays a crucial role in the chemoattractivity of parvalbumins purified from *Rana temporaria.* Nevertheless, the exact function of this ion in parvalbumin chemoattractivity remains to be investigated. We confirmed the importance of calcium in parvalbumin chemoattractivity. In contrast to the calcium-complex parvalbumins, the purified apo-parvalbumins obtained after the EDTA treatment showed no biological activity (Fig. 1.B and Table 4). Moreover, calcium alone is not able to trigger a snake attack. The interaction of ES20 with vomeronasal receptors has been demonstrated to depend on the presence of calcium [22]. We hypothesized that calcium is implicated in the conformational change necessary for the parvalbumin-vomeronasal receptor interaction.

**Table 4:** Bioassays of the calcium-depleted or -supplemented *Osmerus eperlanus* parvalbumins.

Samples	*Thamnophis marcianus*
Complex parvalbumin α/Ca^2+^	3/3
Apo-parvalbumin α	0/2
Complex parvalbumin β/Ca^2+^	2/2
Apo-parvalbumin β	0/5

The chemoattractivity of the purified parvalbumins was assessed according to a standard “all-or-none” snake bioassay based on a non-biological lure coated with the tested sample as in [9]. The test was considered positive if the snake attacked the lure within 20 sec after the first lure-directed tongue flick. The test was considered negative in any other circumstances. A/B corresponds to the number of positive tests/total tests. The purified form of *Osmerus eperlanus* α and β parvalbumins were obtained following the procedure described in [33]. The apo-parvalbumin was prepared by incubating the purified parvalbumin with a calcium-chelating agent (2 mM EDTA) and was then dialysed to remove the EDTA. ND  =  not determined.

### Physiological Role of Parvalbumins in Cutaneous Mucus of Fishes and Amphibians

**Figure 5:**
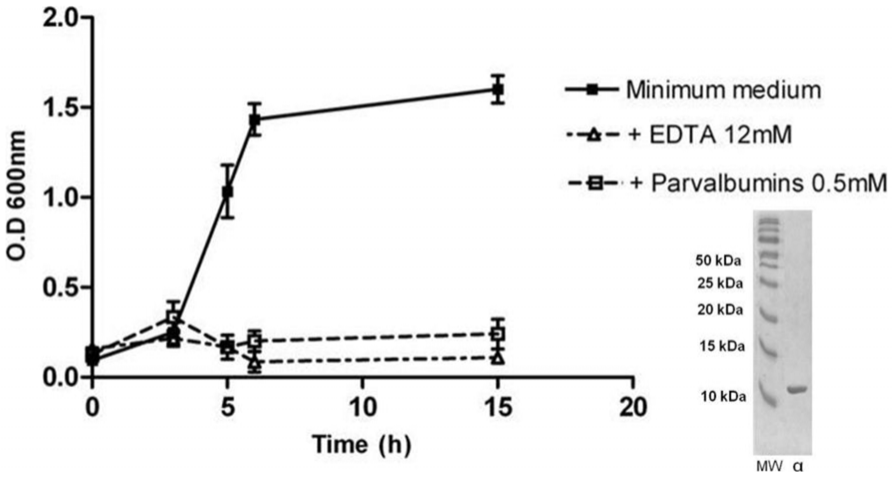
A comparison of *E. coli* growth in the minimum culture medium (filled squares) in the presence of 12 mM EDTA (triangles) or 0.5 mM parvalbumins (squares). All of the results are shown as means ± s.e.m. for three independent replicate experiments per treatment. *Osmerus eperlanus* α parvalbumin was purified according to the procedure described by Gosselin and Rey (1977) (34). SDS-PAGE of purified *Osmerus eperlanus* α parvalbumin.

The intrinsic role of parvalbumins in an unusual location - the cutaneous mucus of amphibians and fish - remains unclear. However, it has been proposed that extracellular calcium-binding proteins, such as calmodulin and parvalbumin, may be involved in controlling the water and ion permeability of skin [27]–[29]. However, we hypothesized that parvalbumins can also participate in innate defense mechanisms against bacteria by chelating divalent cations. To test this hypothesis, *Escherichia coli* growth was measured with and without the presence of purified parvalbumins or EDTA. No bacterial growth was clearly observed in the presence of the parvalbumins (Fig. 5). It is often assumed that there is some form of antibacterial-peptide protection against microorganisms in the cutaneous mucus of both amphibians and fish [30]. We demonstrate here that parvalbumins participate in maintaining an unfavorable medium for bacterial growth through their calcium chelation effect.

## Discussion

In a recent study [9], it was demonstrated that α and β parvalbumins, proteins found in the cutaneous mucus of the frog *Rana temporaria*, elicited prey attacks in *Thamnophis marcianus*. Here we demonstrate that parvalbumins and parvalbumin-like proteins are present in the cutaneous mucus of a frog belonging to another genus (*Lithobates catesbeianus*) and several fish genera from both fresh and salt water environments (*Pimephales promelas*, *Tanichthys albonubes*, *Osmerus erperlanus*). Moreover parvalbumin-like proteins were also found in the cutaneous mucus of an urodele amphibian, *Ambystoma mexicanum* (data not shown), suggesting that these proteins may be widely present among vertebrates with mucus-covered skin such as fishes and amphibians.

All the parvalbumins and parvalbumin-like proteins identified were found to be chemoattractive for the three thamnophiine snakes tested (*Thamnophis marcianus*, *Thamnophis sirtalis* and *Nerodia fasciata*). Moreover, of all the proteins isolated from crude extracts, only parvalbumins showed a chemo-attractive activity. This finding should be interpreted with caution as the denaturing conditions of our protein extraction procedure, semi-preparative SDS-PAGE, are known to disturb protein tertiary structure and thus protein function. Parvalbumins have a relatively high tolerance to denaturation and thus still interact with calcium and thus keep their chemo-attractive properties. As the majority of proteins are altered by these conditions, other putative phagostimulting proteins may have lost their activity during this procedure. Moreover, it seems unlikely parvalbumins are the only chemical cues used by thamnophiine snakes to locate and recognize prey. Other chemical cues are certainly involved in prey recognition and it is possible that our study, focused on the protein skin secretome, did not detect all compounds present in the cutaneous mucus of prey. However, our results do suggest that parvalbumins play a critical role in the chemical prey recognition mechanism of new world natricine snakes. In addition, two old world natricine snakes (*Natrix natrix* and *Natrix maura*) respond to proteinous chemo-attractants from frog skin [7], [9] and brown treesnakes, *Boiga irregularis*, strongly respond to mammalian blood serum fraction known to contain parvalbumins [31], [32]. Taken together, these observations suggest that this parvalbumin-based prey recognition mechanism may also be present in other colubroides snakes.

Parvalbumins are calcium-binding proteins and, as previously reported [9], calcium plays a key role in their chemoattractive properties. However, it has also been clearly established that calcium alone is not able to trigger a snake attack [9]. Nevertheless, the precise role of this ion in the chemoattractivity of parvalbumins remains to be investigated. The interaction of ES20, a chemoattractive protein identified from the mucus of earth worms, with vomeronasal receptors was demonstrated to depend on the presence of calcium [8]. Consequently, it is likely that calcium is also implicated in the conformational change necessary to parvalbumins-vomeronasal receptor interaction.

The intrinsic role of parvalbumins in the cutaneous mucus of amphibians and fishes remains unclear. These proteins are located in many tissues where they typically function as Ca^2+^ buffers [23]–[25]. Before their discovery in the skin secretome of *Rana temporaria*, an extracellular localization of parvalbumins was unknown. However, other calcium-binding proteins have been reported in the mucous layer covering the body of lower vertebrates. For instance, calmodulin has been observed in the cutaneous mucus of three species of fish [28], but its functional significance remains unknown. However, it has been proposed that calmodulin may be involved in the control of the permeability of the skin for water and ions [28]. It is likely that parvalbumins also play such a role, given the crucial function of the skin in active ionic transport fish and amphibians [29]. These proteins could, however, also play a role in the innate defence mechanisms against bacteria. In cutaneous mucus of both amphibians and fishes, protection against microorganisms by antibacterial peptides is often assumed [30]. We hypothesized that parvalbumins could also participate to innate defence mechanisms by chelation of divalent cations necessary for bacterial growth. Our results confirm this hypothesis as bacterial growth is inhibited in presence of parvalbumins. However, as the parvalbumin concentration in the cutaneous mucus is low, we suggest that parvalbumins are primarily implicated in active ionic transport across the skin. Additionally, parvalbumins may help maintain an unfavourable medium for bacterial growth through their calcium chelation effect.

In conclusion, our results demonstrate that parvalbumins and parvalbumin-like proteins are present in the cutaneous mucus of an array of fish and amphibians and play a critical role in the chemical prey recognition mechanisms of natricine snakes.

## Materials and Methods

### Animals

Juvenile specimens of *Lithobates catesbeianus* were obtained from the CDPNE (Comité Départemental de Protection de la Nature et de l’Environnement, Loir et Cher, France). *Pimephales promelas* and *Osmerus eperlanus* specimens were purchased from a commercial dealer. Nine *Thamnophis marcianus* specimens were kindly provided by Prof. G. Toubeau and by the Natural History Museum of Tournai (Belgium). Two *Thamnophis sirtalis* and seven *Nerodia fasciata* specimens were purchased from licensed dealers. All snakes were adults and maintained in the laboratory prior to the beginning of the experiments. The snakes were housed individually in plastic cages (40 cm×25 cm×20 cm) in a common holding room kept on a 12∶12 hr light/dark cycle. Room temperatures ranged from 23 to 27°C. Snakes were fed twice a week on a laboratory-controlled diet composed of fish (*Osmerus eperlanus*), frogs (*Limnonectes macrodon*) and mice (*Mus musculus*). Water was available ad libitum. Prior to tests, snakes were deprived of food for one week. All experiments were approved by the animal ethics committee of the University of Mons. Maintenance and care of animals was in compliance with guidelines of the animal care and use committee.

### Isolation of Proteins from Cutaneous Mucus

Cutaneous mucus extracts were prepared from living *Lithobates catesbeianus*, *Pimephales promelas* and from frozen *Osmerus eperlanus.* Crude extracts were obtained from aqueous washes of the prey according to the procedure described by Leroy et al [9] and Wattiez et al [17] modified as follows. Animals were immersed in distilled water at room temperature (1 ml/g of body weight) for 20 minutes (*Pimephales promelas* and *Osmerus eperlanus*) to one hour (*Lithobates catesbeianus*). After filtration, Dithioerythritol (DTE: 50 mM) and PMSF (2 mM) were added to the washing solutions. Filtered extracts were concentrated by lyophilization and dissolved in 7 mM β-mercaptoethanol (β-MSH). Extracts were then dialyzed three times against 7 mM β-MSH for two hours at 4°C. Dialysed extracts were lyophilized again and stored at −20°C.

Chemoattractive proteins of cutaneous mucus extract were isolated using semipreparative SDS-PAGE as described by Leroy et al. [9]. Briefly, the lyophilized mucus extract was solubilized in Laemmli sample buffer and submitted to SDS-PAGE (T: 15%; C: 3.3%; 7×8.5 cm). A guide strip, cut from the developed gel and stained with Coomassie brilliant blue, made it possible to locate and excise the corresponding protein bands on the unstained part of the gel. Proteins from the excised gel pieces were recovered by pulverizing and soaking them in 50 mM DTE for 24 hours under strong agitation at 4°C. The supernatants were collected, lyophilized and stored at −20°C.

### Western Blot Analysis and Immunohistochemistry

The Ph14 antibodies were obtained from the purified mucus α parvalbumin of *Rana temporaria* [9]. The presence of alpha parvalbumins in cutaneous mucus extracts was ascertained through western blot analysis using anti-α parvalbumin antibodies. Immunohistochemical localization of parvalbumins in skin sections of *Lithobates catesbeianus*, *Pimephales promelas* and *Tanichthys albonubes* was performed using anti-α parvalbumin antibodies. *Tanichthys albonubes,* a fish that does not figure in the normal diet of natricine snakes, was used instead of *Osmerus eperlanus* due to the impossibility of obtaining skin sections from frozen specimens. The paraffin embedded sections were cleared and the sections were blocked with 3% H_2_O_2_ in TBS for 10 min., washed then blocked with Dako Biotin Blocking System (Dako X0590). After washing, they were further blocked with 0.5% casein for 10 min. at room temperature (RT) and incubated firstly with 1/400 antibody for 1 hr at RT, then with biotinylated Goat Anti-Rabbit (Prosan)) at 1/50 for 30 min. at RT, and finally with Strep-ABC complex (Dako, K-0377) at 1/100 for 30 min. at RT. The sections were developed with AEC substrate kit (vector lab, SK-4200) at RT for 20 min., counterstained with Luxol Blue and after drying were mounted with DAKO aqueous mount (Dako, 003181).

The specificity of the different primary antibodies was monitored by pre-incubation with its antigen 10×in excess. For each skin section, no labelling was observed without primary antibodies or in the presence of pre-absorbed primary antibodies.

### Mass Spectrometry Analysis

Excised Protein bands were subjected to in-gel trypsin digestion and analyzed using LC-MS/MS with an HCT ultra-plus ion trap instrument (Bruker) [9]. Briefly, tryptic peptides were reconstituted in 8 µL of loading solvent (acetonitrile 5%, trifluoroacetic acid 0.025% in HPLC-grade water) and 6 µL were loaded onto a precolumn (C18 Trap, 300 µm ID×5 mm, Dionex) using an ultimate 3000 system, delivering a flow rate of 20 µL/min of loading solvent. After desalting for 10 min, the precolumn was switched online with the analytical column (75 µm ID×15 cm PepMap C18, Dionex) equilibrated in 96% solvent A (formic acid 0.1% in HPLC-grade water) and 4% solvent B (acetonitrile 80%, formic acid 0.1% in HPLC-grade water). The peptides were eluted from the precolumn to the analytical column and then to the mass spectrometer with a gradient from 4 to 57% solvent B for 35 min and 57 to 90% solvent B for 10 min, at a flow rate of 300 nL/min delivered by the Ultimate pump. The peptides were analyzed using the “peptide scan” option of the HCT Ultra Ion Trap (Bruker), consisting of a full-scan MS and MS/MS scan spectrum acquisitions in ultrascan mode (26?000 m/z/s). Peptide fragment mass spectra were acquired in data-dependent Auto MS (2) mode with a scan range of 100 to 2800 m/z, three averages, and 5 precursor ions selected from the MS scan from 300 to 1500 m/z. Precursors were actively excluded within a 0.5 minute window, and all singly charged ions were excluded. The peptide peaks were detected and deconvoluted automatically using Data Analysis 2.4 software (Bruker). Mass lists in the form of Mascot Generic Files were created automatically and used as the input for Mascot MS/MS Ion searches of the NCBInr database release 20080704 using an in-house Mascot 2.2 server (Matrix Science). The default search parameters used were: taxonomy = all species, enzyme = trypsin, maximum missed cleavages = 1, fixed modification = Carbamidomethyl (C), variable modification = Oxidation (M), peptide mass tolerance ±1.5 Da, fragment mass tolerance ±0.5 Da, peptide charge = 1+, 2+ and 3+; instrument = ESI-TRAP. Only sequences identified with a Mascot score of at least 50 were considered. For each protein identified from one single peptide, MS/MS spectra were evaluated manually.

### Snake Bioassay

The chemoattractivity of crude mucus extracts and purified proteins was assessed using a standard “all-or-none” snake bioassay [9], [17]. Briefly, a non-biological lure (cooked macaroni, 5 mm diam., 15 mm length) was coated with 30 µl of protein sample or sample buffer (control solution) and placed in the snake’s box (40×25×20 cm) in front of its shelter. The test was considered positive if the snake attacked the lure within 20 sec after the first lure-directed tongue flick. In any other case, the test was considered negative. In each essay, negative (sample buffer) and positive (mucus crude extract of *Rana temporaria*) control tests were carried out. The sample and control solutions were presented in a randomly selected order. In order to determine if the chemoattractivity is due to proteins, protein extracts were treated with proteinase K before being submitted to snake bioassay. The protein extracts were incubated in presence of proteinase K (solubilized in Tris-HCl buffer 50 mM pH 7.5) during 1 hour at 55°C. The action of the proteolytic enzyme was analyzed using SDS-PAGE.

*Nerodia fasciata* bioassays were filmed at 75 frames per second in a glass aquarium (35×17.5×24.5 cm) using four synchronized high speed cameras (Prosilica GE680, VGA CCD camera, Allied Vision Technologies, Stradtroda, Germany) mounted on tripods (in front, on the sides and above the test arena). Illumination during filming came from two cold light spots (Kino Flo Parabeam® 400, 2000 Watt, Burbank, California, USA).

### Anti-bacterial Activity of Parvalbumins

*E. coli* (20 mL of culture in each 50 ml flask) was cultivated at 28°C and 150 rpm in minimum medium 284 containing 0.2% glucose (27). Alpha parvalbumin (0.5 mM) was added to test its potential anti-bacterial activity. Cell growth was monitored by measuring the value of OD_600_ using a Beckman DU-640 spectrophotometer (Beckman).

## Supporting Information

Video S1 The movie shows a positive bioassay with the snake *Nerodia fasciata* attacking a parvalbumin-coated lure after lure-directed tongue flicks. The video was recorded at 75 fps.
(MOV)
